# Evaluation of a single-shot of a high-density viscoelastic solution of hyaluronic acid in patients with symptomatic primary knee osteoarthritis: the no-dolor study

**DOI:** 10.1186/s12891-022-05383-w

**Published:** 2022-05-11

**Authors:** Joan Calvet, Danial Khorsandi, Laura Tío, Jordi Monfort

**Affiliations:** 1 Rheumatology Department Hospital, Institutd’Investigació I InnovacióParcTaulí (I3PT), Universitari Parc Taulí, 08208 Sabadell, Spain; 2 Procare Health Iberia, 08860 Castelldefels, Spain; 3 University of Barcelona, 08007 Barcelona, Spain; 4 IMIM (Institut Hospital del Mar d’Investigacions Mèdiques), Barcelona, Spain; 5 Rheumatology Department Hospital del Mar, 08003 Barcelona, Spain

**Keywords:** Knee osteoarthritis, Hyaluronic acid, Intra-articular injection, WOMAC, High-density viscoelastic gel

## Abstract

**Background:**

Pronolis®HD mono 2.5% is a novel, one-shot, high-density sterile viscoelastic solution, recently available in Spain, which contains a high amount of intermediate molecular weight hyaluronic acid (HA), highly concentrated (120 mg in 4.8 mL solution: 2.5%). The objective of the study was to analyze the efficacy and safety of this treatment in symptomatic primary knee osteoarthritis (OA).

**Methods:**

This observational, prospective, multicenter, single-cohort study involved 166 patients with knee OA treated with a single-shot of Pronolis®HD mono 2.5% and followed up as many as 24 weeks.

**Results:**

Compared with baseline, the score of the Western Ontario and McMaster Universities Arthritis Osteoarthritis Index (WOMAC) pain subscale reduced at the 12-week visit (primary endpoint, median: 9 interquartile range [IQR]: 7–11 versus median: 4; IQR: 2–6; *p* < 0.001). The percentage of patients achieving > 50% improvement in the pain subscale increased progressively from 37.9% (at 2 weeks) to 66.0% (at 24 weeks). Similarly, WOMAC scores for pain on walking, stiffness subscale, and functional capacity subscale showed significant reductions at the 12-week visit which were maintained up to the 24-week visit. The EuroQol visual analog scale score increased after 12 weeks (median: 60 versus 70). The need for rescue medication (analgesics/nonsteroidal anti-inflammatory drugs) also decreased in all post-injection visits. Three patients (1.6%) reported local adverse events (joint swelling) of mild intensity.

**Conclusions:**

In conclusion, a single intra-articular injection of the high-density viscoelastic gel of HA was associated with pain reduction and relief of other symptoms in patients with knee OA.

**Trial registration:**

ClinicalTrial# NCT04196764

**Supplementary Information:**

The online version contains supplementary material available at 10.1186/s12891-022-05383-w.

## Background

**Fig. 1:**
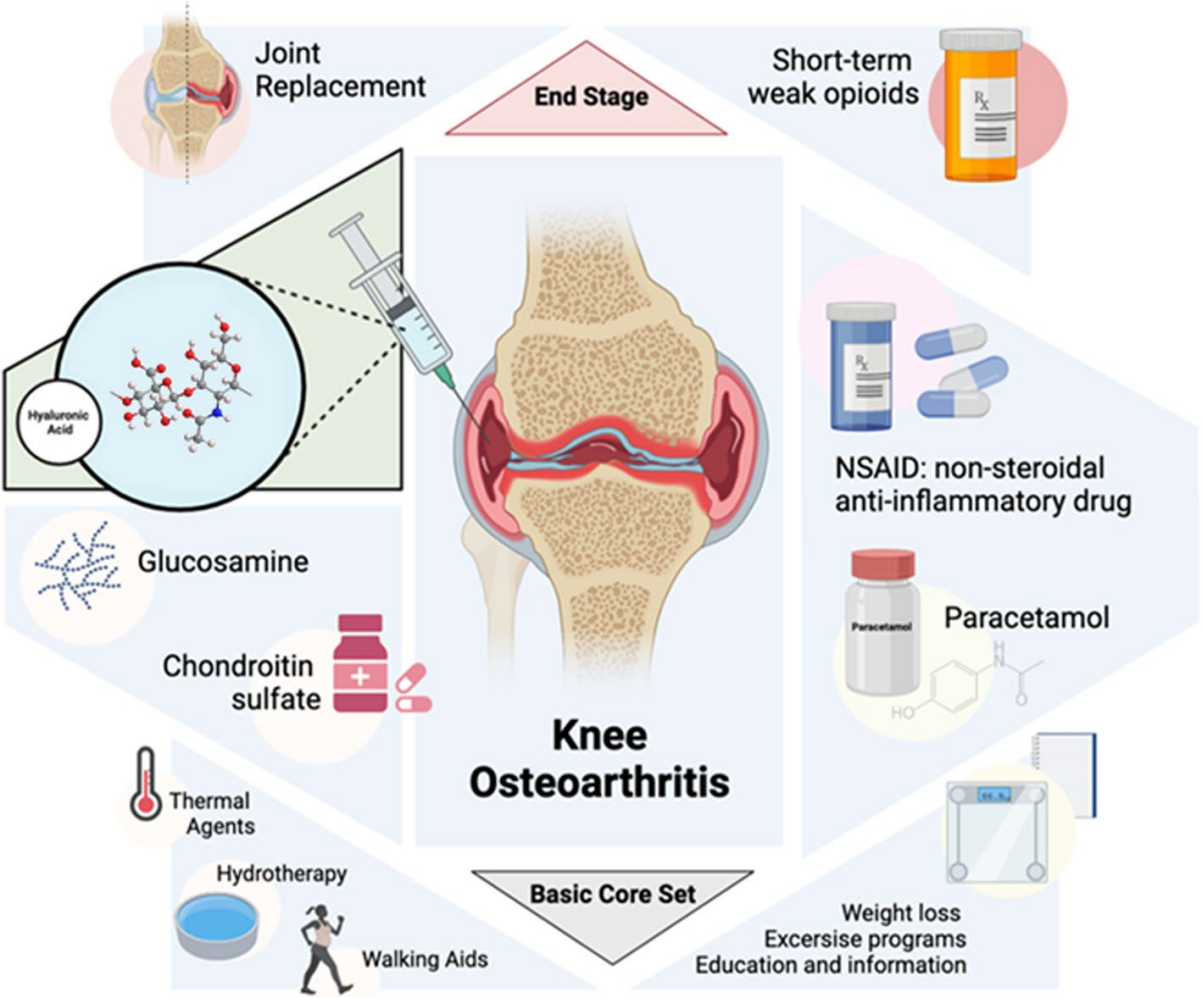
Different available treatments for knee osteoarthritis

Knee osteoarthritis (OA) is one of the most frequent causes of disability in elderly individuals [1]. The prevalence of symptomatic knee OA among men and women aged 60 or over is approximately 10% and 13%, respectively. However, the prevalence is increasing along with the raising of older and obese populations [2]. Knee OA is characterized by diverse pathophysiological changes, including decreased synovial fluid elastoviscosity and hyaluronan concentration [3]. The treatment of knee OA is mainly focused on the control of symptoms, especially pain [4]. Treatment guidelines recommend the use of nonoperative treatments before surgery [2]. The first approach for the relief of symptoms typically involves conservative therapies including exercise, physical therapy, and weight loss [2]. If it fails, analgesics (nonsteroidal anti-inflammatory drugs—NSAIDs -, or acetaminophen), symptomatic slow action drugs for osteoarthritis (SYSADOA; such as glucosamine, chondroitin), or intra-articular therapies (corticosteroids, hyaluronic acid -HA-, platelet-rich plasma) are commonly prescribed [5]. If none of those treatments work, joint replacement would be the final solution. Unfortunately, to date, there are no approved disease-modifying drugs for OA [6]. Although effective for pain relief, analgesics and NSAIDs are accompanied by adverse effects (AEs) [7]. A summary of different treatments for knee osteoarthritis is provided in Fig. 1.

Regarding intra-articular injections with HA, they are getting attraction for the treatment of knee OA. Diverse randomized clinical trials have demonstrated the efficacy and safety of HA for the relief of symptoms in knee OA [8–10]. Bellamy et al., [8] in a Cochrane systematic review, evaluated the effects of diverse HA products for the treatment of knee OA in 76 randomized, controlled clinical trials. Pooled analyses versus placebo highlighted the efficacy of HA injections on pain, patient global assessment, and function after the injection, especially at the 5 to 13-week. Overall, the efficacy of HA injections was comparable with NSAIDs; however, the effect of HA injections was more durable than intra-articular corticosteroids. The HAs are frequently classified according to their molecular weight (MW): low (500–730 kDa), intermediate (800–2,000 kDa), or high (2,000–6,000 kDa) [11]. High-MWHAs have been associated with greater anti-inflammatory and proteoglycan synthesis effects, viscoelasticity maintenance, and joint lubrication. Comparative studies have revealed a greater improvement for pain relief with high-MWHAs than low-MWHAs and intra-articular placebo [12, 13]. In addition, high-MWHAs have been correlated to a higher percentage of AEs. Accordingly, injection site flare-ups with swelling, pain, and increased warmth [12, 14]. Overall, to minimize the risk of AEs, there is still a need for optimizing the HA treatment toward being single-shot and having a longer duration of action at the same time.

Pronolis®HD mono 2.5% (Procare Health, Spain/KD Intra-Articular® Gel 2.5%) is a novel, one-shot, high-density sterile viscoelastic solution, recently marketed in Spain, which contains a high amount of intermediate-MWHA, highly concentrated (120 mg in 4.8 mL solution: 2.5%) [15–17].

The objective of the present study was to analyze the efficacy and safety of a single-shot of this novel high-density viscoelastic gel of HA for the treatment of symptomatic primary knee OA.

## Methods

### Study design

This observational, prospective, multicenter, single-cohort study involved patients with knee OA who initiated treatment with Pronolis®HD mono 2.5% following manufacturer instructions (No-dolor study; ClinicalTrial# NCT04196764). A total of 29 healthcare centers from Spain (including Services of Rheumatology, Traumatology, Sports Medicine, and Pain Medicine) participated in the study. Main inclusion criteria were: adult men and women (aged over 18); with the diagnosis of primary knee OA, according to American College of Rheumatology criteria [18]; having performed a radiographic assessment of knee OA within the previous 18 months to study inclusion; showing a visual analog scale (VAS) score for pain ≥ 4 (out of 10) at study inclusion; having started treatment with Pronolis®HD mono 2.5% (prescribed as part of routine clinical practice) at the time of the study inclusion; and signing informed consent. Main exclusion criteria were: patients with intolerance to HA; hypersensitivity to intra-articular injections; infection in the knee joint; skin disorders or infections, either at the injection site or systemic; coagulation disorders that contraindicate the injection; prescription of intra-articular injections in both knees; diagnosis of autoimmune rheumatic diseases, connective tissue conditions, or microcrystalline disorders; and history of traumas in the knee joint; previous surgery in the knee joint. The complete list of inclusion and exclusion criteria is shown in Supplementary Table 1. The treatment consisted of a single-shot of Pronolis®HD mono 2.5% [14].

### Endpoints and Measures

Patients were followed up for as many as 24 weeks. Visits were scheduled after 2, 4, 12, and 24 weeks of the injection. Visits were undergone in-person (face-to-face) at the hospital, except the 2-week visit that consisted of a phone call. The primary endpoint included the change of scores in the pain subscale from the Spanish version of the Western Ontario and McMaster Universities Arthritis Osteoarthritis Index (WOMAC) at the 12-week visit, compared with baseline. The minimal perceptible clinical improvement (MPCI) was analyzed by following the Ehrich et al. methodology [19]. OMERACT-OARSI rate of responders was calculated according to Pham et al. [20]. Secondary endpoints comprised the evaluation of pain (item evaluating pain on walking), joint stiffness, and functional capacity (by WOMAC subscales), the quality of life of patients, the need for rescue medication (analgesics/NSAIDs), the satisfaction with the treatment, and the development of AEs during the study period. Each WOMAC item was assessed with a 0–4 Likert scale, where 0 represented “none” and 4 indicated “very much”. The patient’s quality of life was determined at the 12-week visit by using the EuroQol-5D-5L (EQ-5D-5L) questionnaire [21]. The EQ-VAS is scored between 0 (the worst patient’s self-rated health) and 100 (the best). Satisfaction with the treatment was analyzed at all post-injection visits with a 1–5 Likert scale, where 1 represented “very satisfied” and 5 indicated “very unsatisfied”. Only patients with available data at the 12-week visit were considered for the efficacy analysis.

### Determination of the sample size

The primary objective included score changes in the WOMAC pain subscale, from baseline. Studies by Zhang et al. [22] and Pavelka et al. [23] assumed 20 mm and 21 mm as a standard deviation (SD) of change, respectively, on a 100 mm VAS. Furthermore, Raynauld et al. [24] reported a mean change of -4.4 after 12 months of treatment, compared to baseline, using a Likert scale, and an SD of 3.9 (equivalent to 19.4 on a 100 mm VAS). Calculations estimated that a sample size of 270 patients could provide a precision of ± 2.4 mm (on 0–100 scale) for the change, with a 95% confidence interval (95%CI, confidence interval for one mean), an alpha of 0.05, and assuming an SD of 20 mm. Considering 10% of the loss to follow-up, the estimated number of patients to be recruited was 300. These estimations were carried out with PASS software (2011 version). Given the lack of available participants, the period of recruitment was extended from eight months (initially planned in the protocol) to one year and 10 months.

### Statistical analysis

Categorical variables were expressed as absolute and relative frequencies; whereas continuous ones with the mean, SD, median, 95%CI, or interquartile range (IQR, i.e. percentile 25–75). Comparisons in variables between baseline and post-injection visits were performed using the paired-samples Student’s t-test or Wilcoxon test, when appropriate. Linear mixed models were used to calculate the estimation (95%CI) of the effect of the studied variables in the primary variable (WOMAC A at 12-week visit). Statistical significance was established when *p* < 0.05. All statistical procedures were carried out with SAS 9.4 software.

## Results

### Patient characteristics at baseline

**Fig. 2:**
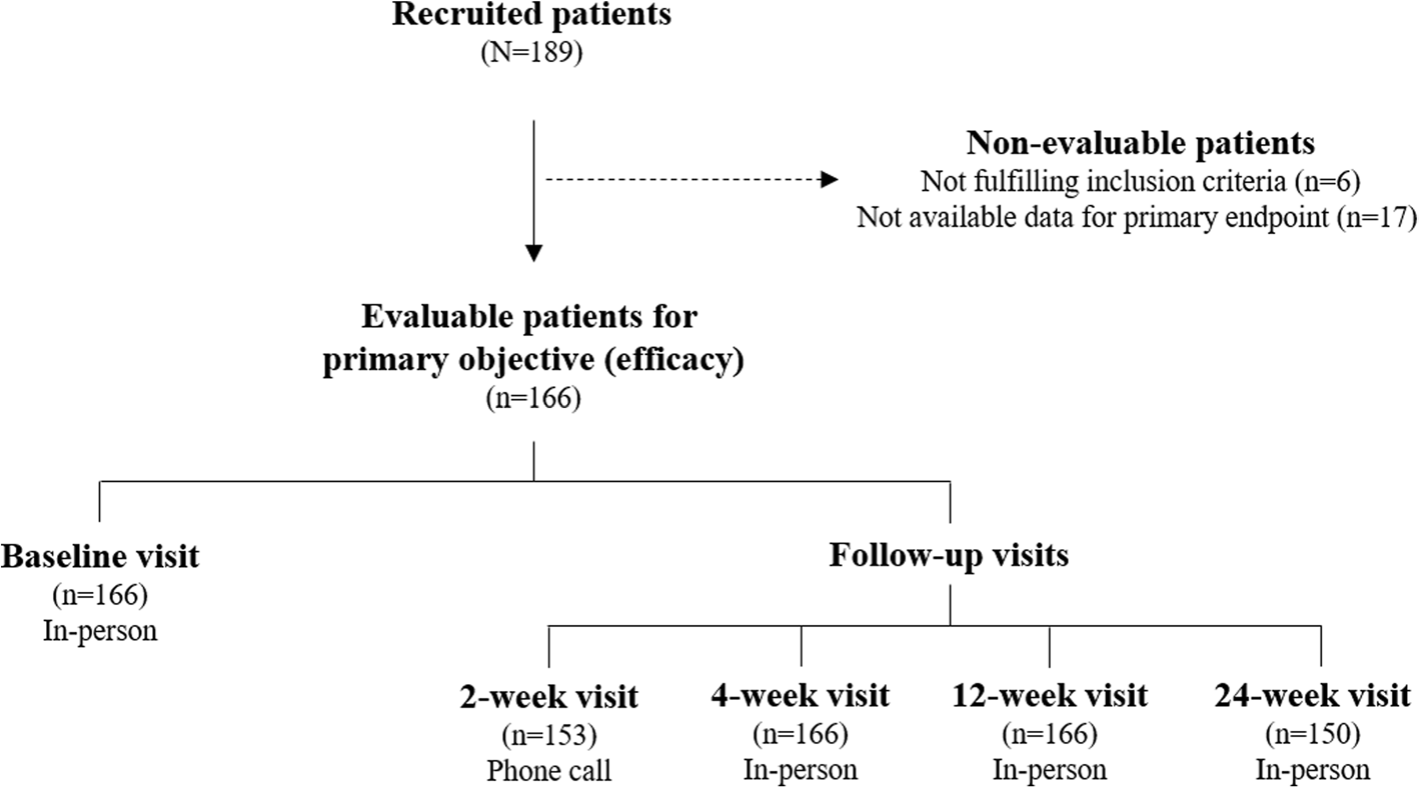
Flowchart of patients and design of the study

A total of 189 patients were initially recruited; however, 23 were not evaluable for the primary objective (Fig. 2). Patients were predominantly females (75.9% of total) and aged over 60 years (66.9%). Baseline sociodemographic and clinical characteristics of patients are shown in Table 1. Their mean age was 63.2 years (SD: 11.1). Considering the body mass index, 49.3% were overweight (25—< 30 kg/m^2^), 34.5% were obese (≥ 30 kg/m^2^), and 16.2% were normal weight (< 25 kg/m^2^). The mean time from the diagnosis of knee OA to the current HA treatment was 7.2 years (SD: 7.5). Regarding pain, 12.0% of patients considered to control adequately the pain, whereas 79.5% indicated that the treatment, followed to that time (baseline), was not enough for controlling the pain and living a normal life. A total of 150 patients (90.4%) completed the study, however 16 (9.6%) did not so. Information about the early discontinuation was available in seven of them (six due to lost to follow-up and one due to patient decision).

**Table 1:** Baseline sociodemographic and clinical characteristics of patients

	**Total patients** (*N *= 166)
Age, mean years (SD)	63.2 (11.1)
Gender, n (%)	
Male	40 (24.1)
Female	126 (75.9)
Body mass index, mean Kg/m^2^ (SD)	28.6 (4.3)
Time from OA diagnosis to treatment, mean years (SD)	7.2 (7.5)
Type of primary knee OA, n (%) ^*^	
Patellofemoral	70 (42.4)
Femorotibial	95 (57.6)
Radiological grading (Kellgren-Lawrence classification), n (%)	
Grade 2 (Mild)	39 (23.5)
Grade 3 (Moderate)	113 (68.1)
Grade 4 (Severe)	14 (8.4)
Previous intra-articular injections for knee OA, n (%)	
No	103 (62.0)
Yes	63 (38.0)
Type of injection, n (%)	
Corticosteroids	50 (79.4)
HA	23 (36.5)
Time since last corticosteroid injection, mean months (SD)	38.8 (48.1)
Time since last HA injection, mean months (SD)	26.4 (23.4)

SD, standard deviation; OA, osteoarthritis; HA, hyaluronic acid

^*^ Information from one patient was missing

### WOMAC results

**Fig. 3:**
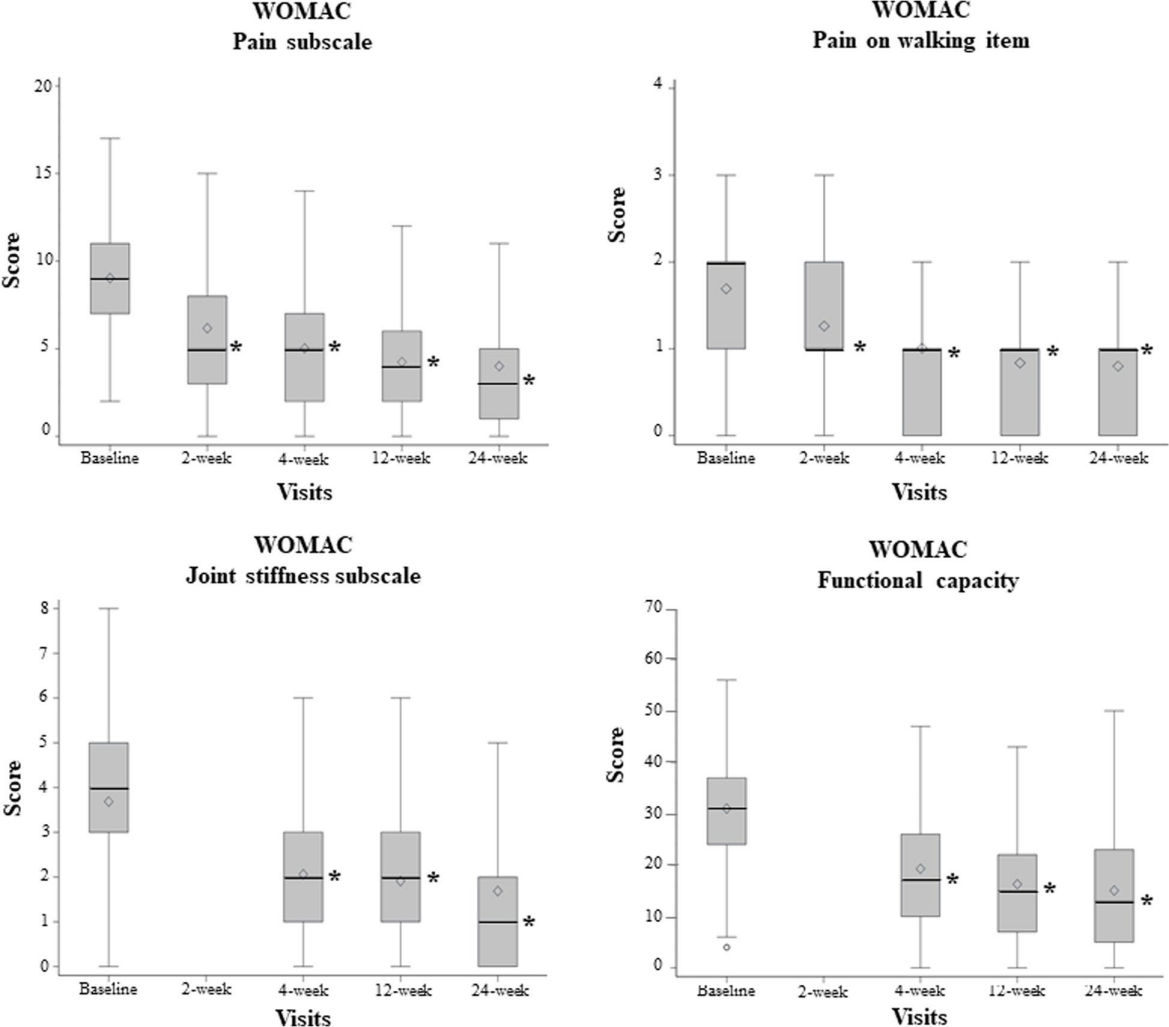
Boxplots showing the evolution of WOMAC scores WOMAC, Western Ontario and McMaster Universities Osteoarthritis Index The rhombus shows the mean value of the respective subscale/item. Asterisks represent statistical differences (Wilcoxon test) found with respect to baseline (*p* < 0.001). Data for WOMAC joint stiffness and functional capacity subscales at the 2-week visit were no collected.

Compared with baseline, the score of the WOMAC pain subscale was reduced at the 12-week visit (primary endpoint) in 4.78 points (95%CI: -5.4;—4.12) representing a mean relative reduction of 48.2% (95%CI: 41.4–55.0). No association was observed between this improvement and other variables relevant to WOMAC score (Supplementary Table 2). The median observed score for this variable was: 9 (IQR: 7–11) at the baseline visit versus 4 (IQR: 2–6) at the 12-week visit (Table 2 and Fig. 3). The percentage of patients achieving > 50% improvement in the pain subscale (with respect to baseline) increased from the 2-week (37.9%) to the 4-week (52.4%), 12-week (61.4%), and 24-week visits (66.0%; Supplementary Fig. 1). Similarly, scores for pain on walking item, stiffness subscale, and functional capacity subscale showed reductions at the 12-week visit (*p* < 0.001 in all; Table 2 and Fig. 3). Mean relative reductions were 47.1% (95%CI: 38.9–55.4), 45.9% (95%CI: 39.0–52.9), and 42.4% (95%CI: 36.0–48.9), respectively. A total of 61.5% of patients (for pain on walking item), 74.7% (for joint stiffness subscale), and 84.8% (for functional capacity subscale) achieved an improvement in WOMAC scores at the 12-week visit, compared with baseline. Furthermore, the clinical benefit in WOMAC pain, stiffness, functional capacity subscales, and pain on walking item was maintained up to the 24-week visit (Table 2). The complete information about efficacy outcomes, relative reductions, and change in WOMAC result at all post-injection visits compared with baseline are shown in Supplementary Tables 3-5. The treatment achieved a MPCI in all WOMAC subscales at the 12-week visit (Supplementary Table 6). The OMERACT-OARSI rate of responders at the 12-week visit was 72.9%.

**Table 2:** Efficacy endpoint results at the 12-and 24-week visits

	**Baseline**	**12-week visit**	Mean absolute difference	**p** ^*^	**24-week visit**	Mean absolute difference	p ^*^
N	166	166			150		
WOMAC questionnaire							
Pain subscale							
Median score (IQR)	9 (7–11)	4 (2–6)	5 (2–7)	< 0.001	3 (1–5)	5 (2–8)	< 0.001
Mean score (SD)	9.0 (3.5)	4.3 (3.4)	4.8 (4.3)		4.0 (3.8)	5.0 (4.6)	
95%CI	8.5–9.6	3.7–4.8	4.1–5.4		3.4–4.6	4.3–5.8	
Pain on walking							
Median score (IQR)	2 (1–2)	1 (0–1)	1 (0–2)	< 0.001	1 (0–1)	1 (0–2)	< 0.001
Mean score (SD)	1.7 (1.0)	0.8 (0.8)	0.9 (1.1)		0.8 (0.9)	0.9 (1.1)	
95%CI	1.5–1.8	0.7–1.0	0.7–1.0		0.7–0.9	0.7–1.0	
Stiffness subscale							
Median score (IQR)	4 (3–5)	2 (1–3)	2 (0–3)	< 0.001	1 (0–2)	2 (1–3)	< 0.001
Mean score (SD)	3.7 (1.7)	1.9 (1.4)	1.8 (2.0)		1.7 (1.7)	2.0 (2.2)	
95%CI	3.4–4.0	1.7–2.1	1.5–2.1		1.4–2.0	1.6–2.3	
Functional capacity subscale							
Median score (IQR)	31 (24–37)	15 (7–22)	14 (4–25)	< 0.001 ^**^	13 (5–23)	14 (5–29)	< 0.001 ^**^
Mean score (SD)	31.0 (12.7)	16.3 (11.8)	14.7 (14.5)		15.1 (12.5)	16.1 (15.4)	
95%CI	29.1–33.0	14.5–18.1	12.4–16.9		13.0–17.1	13.6–18.6	
EQ-5D-5L							
Mobility							
Median score (IQR)	3 (2–3)	2 (1–3)	1 (0–1)	< 0.001	NA	NA	NA
Mean score (SD)	2.8 (0.9)	2.1 (0.8)	0.8 (0.9)		NA	NA	
95%CI	2.7–2.9	1.9–2.2	0.6–0.9		NA	NA	
Self-care							
Median score (IQR)	2 (1–3)	1 (1–2)	0 (0–1)	< 0.001	NA	NA	NA
Mean score (SD)	2.2 (1.1)	1.6 (0.8)	0.6 (0.9)		NA	NA	
95%CI	2.1–2.4	1.5–1.8	0.5–0.7		NA	NA	
Usual activities							
Median score (IQR)	3 (2–3)	2 (1–2)	1 (0–1)	< 0.001	NA	NA	NA
Mean score (SD)	2.7 (0.9)	2.0 (0.9)	0.8 (1.0)		NA	NA	
95%CI	2.6–2.8	1.8–2.1	0.6–0.9		NA	0.6–0.9	
Pain/discomfort							
Median score (IQR)	3 (3–4)	2 (2–3)	1 (0–2)	< 0.001	NA	NA	NA
Mean score (SD)	3.0 (0.8)	2.2(0.8)	0.9 (1.0)		NA	NA	
95%CI	2.9–3.2	2.0–2.3	0.7–1.0		NA	NA	
Anxiety/depression							
Median score (IQR)	2 (1–3)	1 (1–2)	0 (0–1)	< 0.001	NA	NA	NA
Mean score (SD)	2.1 (1.1)	1.5 (0.8)	0.6 (1.1)		NA	NA	
95%CI	1.9–2.3	1.4–1.6	0.5–0.8		NA	NA	
EQ VAS							
Median score (IQR)	60 (40–75)	70 (60–85)	10 (0–30)	< 0.001	NA	NA	NA
Mean score (SD)	55.4 (22.4)	69.2 (19.7)	13.8 (23.6)		NA	NA	
95%CI	52.0–59.0	66.2–72.2	10.1–17.4		NA	NA	
Consumption of analgesics/NSAIDs as rescue medication, n (%) patients	120 (72.3)	63 (38.0)	NA	< 0.001^***^	58 (38.7)	NA	< 0.001^***^

WOMAC, Western Ontario and McMaster Universities Osteoarthritis Index; IQR, interquartile range (percentile 25–75); SD, standard deviation; 95%CI, confidence interval; VAS, visual analogue scale; NA, not available; NSAIDs, nonsteroidal anti-inflammatory drugs

^*^ If not indicated otherwise, the statistical analysis was Wilcoxon test, ^**^paired t test, ^***^ McNemar test

### Quality of life, rescue medication and patient satisfaction results

**Fig. 4:**
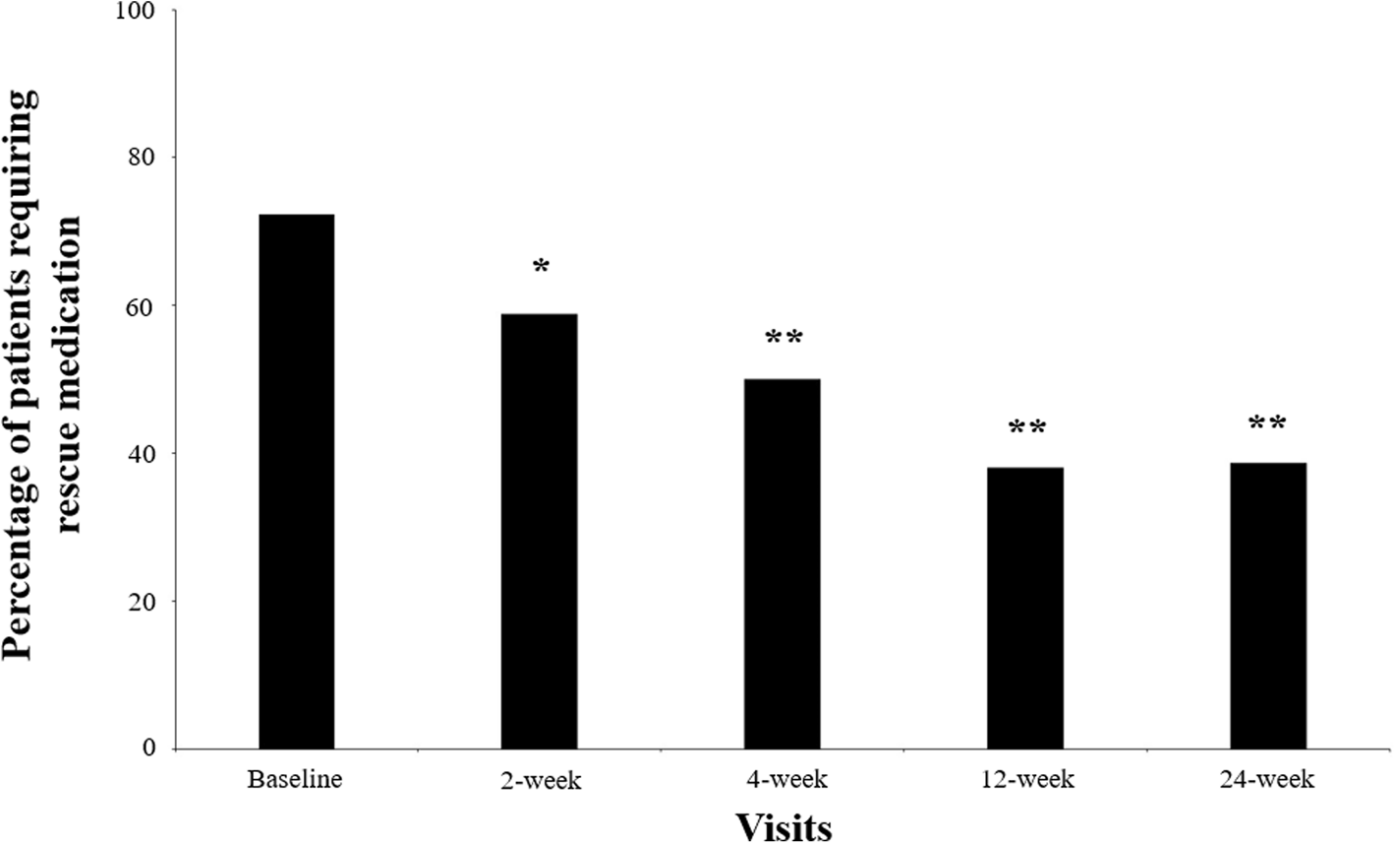
Evolution of need for rescue medication during the post-injection visits McNemar test: ^*^ *p* = 0.007, ^**^ *p* < 0.001

Scores from all dimensions in EQ-5D-5L reduced from baseline to the 12-week visit: mobility (median: 3, IQR: 2–3 at baseline versus median: 2, IQR: 1–3 at the 12-week visit; *p* < 0.001), self-care (median: 2, IQR: 1–3 versus median: 1, IQR: 1–2; *p* < 0.001), usual activities (median: 3, IQR: 2–3 versus median: 2, IQR: 1–2; *p* < 0.001), pain/discomfort (median: 3, IQR: 3–4 versus median: 2, IQR: 2–3; *p* < 0.001), and anxiety/depression (median: 2, IQR: 1–3 versus median: 1, IQR: 1–2; *p* < 0.001). Similarly, the EQ-VAS score increased after 12 weeks (median: 60, IQR: 40–75 versus median: 70, IQR: 60–85; *p* < 0.001). The need for rescue medication (analgesics/NSAIDs) also decreased in all post-injection visits (*p* = 0.007 at the 2-week and *p* < 0.001 at the remaining visits), compared with baseline (Fig. 4). Of patients, 56.6% were very satisfied or satisfied with the treatment at the 2-week visit. This percentage increased to 64.5%, 79.5%, and 82.7% at the 4-, 12-, and 24-week visits, respectively. Median scores for satisfaction were 2 (IQR: 2–3) in all post-injection visits.

### The safety profile of the treatment

Three patients (1.6%) reported 4 local AEs (joint swelling, *n* = 3; and ligament sprain, *n* = 1). None of the AEs was serious, and all of them were mild in severity. A total of 150 patients (90.4%) completed the study; however, 16 did not so. Loss to follow-up was the reason for early withdrawal in 6 out of 7 patients with available information (85.7%).

## Discussion

In our study, the use of a novel high-density viscoelastic gel of HA has been associated with a clinical efficacy (in terms of pain relief) for patients with symptomatic knee OA. This benefit was achieved early (two weeks after the injection) and maintained for, at least, 24 weeks. In general, clinical improvement has been established with a minimum difference of 20% in efficacy outcomes [25], like scores in the WOMAC questionnaire. With the cautelous of involving different methodologies and HA gels among studies, our results agree with the literature implicating diverse HA products [8–10, 12, 26, 27]. For instance, Berenbaum et al., [24] in a randomized, double-blind, controlled trial compared the efficacy of 3-weekly injections between an intermediate- versus a low-MWHA in 426 patients with symptomatic knee OA. The decrease in WOMAC pain score at the 24-week (after the end of treatment) was significantly greater with the intermediate-MWHA preparation (mean: 22.9 mm, SD: 1.4) than the low one (mean: 18.4, SD: 1.5 mm). Moreover, the proportion of responders (OMERACT-OARSI criteria) with the intermediate-MWHA was also significantly higher (73% versus 58%). The percentage of patients reporting AEs was similar in both groups (35.2% versus 33.2%). Raman et al., [27] in a prospective, randomized, clinical trial compared the effectiveness of a high- versus low-MWHA in 392 patients with knee OA. Compared with baseline, scores in the WOMAC pain subscale at the 24-week post-injection visit were significantly lower with the high-MWHA (mean: 9.2 versus 5.1) than with the low one (mean: 8.8 versus 8.3). Nevertheless, the number of patients suffering from treatment-related AEs was higher with the high-MWHA (*n* = 39) than the low one (*n* = 30). In fact, one patient receiving the high- MWHA experienced a serious AE (pseudo-sepsis in the knee) and required hospitalization [27]. Our study also revealed the efficacy of the high-density viscoelastic gel of HA regarding pain on walking, joint stiffness, functional capacity, and quality of life, in agreement with previous studies [8–10, 12, 24]. Raynauld et al. [24], in a prospective, randomized, multicenter study compared the effectiveness of a high-MWHA product versus conventional care in 255 patients with knee OA. Changes at 12-month post-injections were significantly greater for the high-MWHA in WOMAC pain score (-38.4% versus -13.3% with conventional care), stiffness (-34.7% versus -10.4%), and physical function (-31.4% versus -14.5%). Nonetheless, the percentage of patients experiencing AEs was numerically greater with high-MWHA (96%) than conventional care (90%).

Overall, the efficacy of high-MWHA has been demonstrated to be superior to intermediate- and low-MWHA. Altman et al., [12] in a meta-analysis determined the efficacy of HA products according to their MW in 11 randomized clinical trials and 2,094 patients. Pooled efficacy results revealed a greater pain relief for high-MWHA (effect size: -0.52, 95%CI: -0.56 to -0.48) than intermediate- (effect size: -0.31, 95%CI: -0.42 to -0.20) and low-MWHA (effect size: -0.18, 95%CI: -0.19 to -0.17). By contrast, the percentage of AEs is also higher in products with high-MWHA [26, 27]. Reichenbach et al., [28] in a systematic review and meta-analysis, revealed a double risk for local AEs and post-injection flares with high-MW, cross-linked HA formulations than with intermediate- or low-MWHA preparations. In addition, pseudosceptic reactions (granulomatous inflammation of the synovium) have been reported in few cases, especially with cross-linked formulations of the highest-MWHA [9, 29]. In our study, only mild AEs were reported and were predominantly resolved within few days.

On the other hand, most HA preparations implicate between 3 and 5 injections, nevertheless, there are cross-linked formulations that require a single-shot, delivering the same HA dose as multi-injection preparations. Petterson et al., [30] in a multicenter, double-blind, randomized, placebo-controlled trial demonstrated the superior success rate (≥ 50% improvement and ≥ 20 mm absolute improvement, concerning baseline, in WOMAC pain subscale at the 26-week) with a 4-mL single HA injection than with saline. The clinically meaningful reduction of pain was evidenced within the 2-week post-injection. Although studies specifically designed are required, single-shots formulations of HA may also contribute to minimizing the risk for the development of AEs.

In our opinion, the notable results obtained in our study (presumably associated with the HA), especially the 48% reduction in WOMAC pain subscale, 66.0% of patients achieving more than 50% improvement in pain subscale, the early initiation of the clinical benefit (within 2 weeks), and its maintenance for a long-term period of time (24 weeks, at least), are greater than expected by an intermediate-MWHA preparation. These superior results might be associated with the high concentration and amount of HA, and thus to the high-density of the viscoelastic gel. In HA-based aqueous solutions, higher concentrations of HA are correlated (linear relationship) with higher densities of the viscoelastic solution [31, 32]. Therefore, it could be hypothesized that high-density viscoelastic gels of HA provide higher efficacy (similar to high-MWHA) while avoiding the higher incidence of AEs. To our knowledge, to date, none of the studies have specifically evaluated the value of HA products considering the density of the viscoelastic gel.

The main limitation of our study was the absence of a control or comparator group. Although the treatment of OA has an important placebo effect [33], and acknowledging that a control group would have strengthened the conclusions, results are in concordance with controlled studies, revealing a superior impact of HA injections. Another limitation was related to the limited sample size of the study (*n *= 166), not fulfilling the estimated one in the protocol (*n* = 300). Despite extending considerably the recruitment period, the availability of patients was insufficient. Yet, results found in our study are in concordance with other HA products [8–10, 12, 26, 27].

## Conclusions

In conclusion, a single intra-articular injection of the high-density viscoelastic gel of HA was associated with pain reduction and relief of other symptoms in patients with knee OA. Further long-term studies, with a larger cohort of patients, and head-to-head non-inferiority analyses should be performed to corroborate the present results.

## Authors' contributions

All authors contributed to the study conception and design, material preparation, data collection and analysis. All authors commented on previous versions of the manuscript and read and approved the final manuscript.

## Supplementary Information

**Additiona file 1:Supplementary Figure 1.** Improvement in the painsubscale score of the Western Ontario and McMaster Universities ArthritisOsteoarthritis Index (WOMAC) at different post-injection visits with respect tothe baseline. **Supplementary Table1**. Inclusion and exclusioncriteria. **Supplementary Table2.** Linear mixed models fromvisit effect on WOMAC A absolute change, considering patient effect. **Supplementary Table 3**. Efficacy outcomesduring all post-injection visits. **Supplementary Table 4**. Relative reductions of WOMAC results at allpost-injection visits compared with baseline. **Supplementary Table5**. Changein WOMAC results at all post-injection visits compared with baseline. **Supplementary Table6**. Calculations to analyze theminimal perceptible clinical improvement** with the treatment at the 12-week visit

## Data Availability

The dataset(s) supporting the conclusions of this article is(are) included within the article (and its additional file(s)).

## Abbreviations

HA: Hyaluronic acid
IQR: Interquartile Range
MW: Molecular weight
NSAIDs: Nonsteroidal anti-inflammatory drugs
OA: Osteoarthritis
WOMAC: The Western Ontario and McMaster Universities Osteoarthritis Index
