# Supplementary material for: Evaluation of a single-shot of a high-density viscoelastic solution of hyaluronic acid in patients with symptomatic primary knee osteoarthritis: the no-dolor study
Source: BMC Musculoskelet Disord. 2022 May 11;23:442. doi: 10.1186/s12891-022-05383-w (PMC9097347; doi:10.1186/s12891-022-05383-w)
Supplement: Supplementary file 1 — Additiona file 1:Supplementary Figure 1. Improvement in the painsubscale score of the Western Ontario and McMaster Universities ArthritisOsteoarthritis Index (WOMAC) at different post-injection visits with respect tothe baseline. Supplementary Table1. Inclusion and exclusioncriteria. Supplementary Table2. Linear mixed models fromvisit effect on WOMAC A absolute change, considering patient effect. Supplementary Table 3. Efficacy outcomesduring all post-injection visits. Supplementary Table 4. Relative reductions of WOMAC results at allpost-injection visits compared with baseline. Supplementary Table5. Changein WOMAC results at all post-injection visits compared with baseline. Supplementary Table6. Calculations to analyze theminimal perceptible clinical improvement** with the treatment at the 12-week visit [file 12891_2022_5383_MOESM1_ESM.docx]

**Supplementary Figures legends**

**Supplementary Figure 1**. Improvement in the pain subscale score of the Western Ontario and McMaster Universities Arthritis Osteoarthritis Index (WOMAC) at different post-injection visits with respect to the baseline.

**Supplementary Table 1**. Inclusion and exclusion criteria

| **Number** | **Inclusion criteria** |
| --- | --- |
| 1 | Adult men and women (aged over 18) |
| 2 | Diagnosis of primary knee osteoarthritis (OA), according to American College of Rheumatism criteria |
| 3 | Having performed a radiographic assessment (X-ray) of knee OA within the previous 18 months to study inclusion |
| 4 | Assisting to consultation with the specialist for the control of the symptomatic knee OA |
| 5 | Showing a visual analog scale score for pain ≥4 (out of 10) at study inclusion |
| 6 | Having started the treatment with Pronolis® HD mono 2.5% (prescribed as part of routine clinical practice) |
| 7 | Being able to understand and complete the questionnaires (not having a cognitive impairment that complicates carrying out such activities) |
| 8 | Signing an informed consent |
|  |  |
| **Number** | **Exclusion criteria** |
| 1 | Patients with intolerance to hyaluronic acid |
| 2 | Patients with hypersensitivity to the intra-articular injections |
| 3 | Patients with infection in the knee joint |
| 4 | Patients with skin disorders or infections, either at the injection site or systemic |
| 5 | Patients with coagulation disorders that contraindicate the injection |
| 6 | Patients with prescription of intra-articular injections in both knees |
| 7 | Patients with diagnosis of autoimmune rheumatic diseases, connective tissue conditions, or microcrystalline disorders |
| 8 | Patients with history of traumas in the knee joint |
| 9 | Patients with previous surgery in the knee joint |
| 10 | Pregnant or lactating women |

**Supplementary Table 2.** Linear mixed models from visit effect on WOMAC A absolute change, considering patient effect.

| **Predictors** | **Estimation** | **95% CI WOMAC A** | ***p*** |
| --- | --- | --- | --- |
| **With adjustment variables** | | | |
| (Intercept) | 6.36 | 4.00 – 8.71 | -- |
| Visit | -4.78 | -5.44 – -4.12 | <0.0001 |
| **Age** | 0.04 | 0.01 – 0.08 | 0.03 |
| (Intercept) | 8.88 | 8.22 – 9.53 | -- |
| Visit | -4.78 | -5.44 – -4.12 | <0.0001 |
| **Time (years) since the diagnosis** | 0.02 | -0.03 – 0.08 | 0.45 |
| (Intercept) | 8.44 | 7.55 – 9.33 | -- |
| Visit | -4.78 | -5.44 – -4.12 | <0.0001 |
| **Sex (feminine vs masculine)** | 0.77 | -0.17 – 1.72 | 0.11 |
| (Intercept) | 8.74 | 8.02 – 9.45 | -- |
| Visit | -4.78 | -5.44 – -4.11 | <0.0001 |
| **Type of primary knee osteoarthrosis (femorotibial vs femoropatellar)** | 0.52 | -0.31– -1.35 | 0.22 |
| (Intercept) | 8.93 | 8.03 – 9.83 | -- |
| Visit | -4.78 | -5.44– -4.12 | <0.0001 |
| **Radiologic grade** (Moderate to mild) | -0.04 | -1.01 – 0.93 | 0.12 |
| **Radiologic grade** (Severe to mild) | 1.50 | -0.13 – 3.12 |  |
| (Intercept) | 9.40 | 8.67 – 10.14 | -- |
| Visit | -4.78 | -5.44 – -4.12 | <0.0001 |
| **Previous intraarticular treatment of primary knee osteoarthritis (no vs yes)** | -0.61 | -1.44 – 0.23 | 0.16 |
| (Intercept) | 8.89 | 8.34 – 9.43 | -- |
| Visit | -4.78 | -5.44 – -4.12 | <0.0001 |
| **Previous NSAIDs consumption (frequently+ diary vs never+ occasionally)** | 0.99 | -0.18 – 2.17 | 0.10 |

95%CI, 95% confidence interval

**Supplementary Table 3**. Efficacy outcomes during all post-injection visits

|  | **Baseline** | **2-week visit** | **p ^*^** | **4-week visit** | **p ^*^** | **12-week visit** | **p ^*^** | **24-week visit** | **p ^*^** |
| --- | --- | --- | --- | --- | --- | --- | --- | --- | --- |
| N | 166 | 153 |  | 166 |  | 166 |  | 150 |  |
| **WOMAC questionnaire** |  |  |  |  |  |  |  |  |  |
| Pain subscale |  |  |  |  |  |  |  |  |  |
| Median score (IQR) | 9 (7-11) | 5 (3-8) | <0.001 | 5 (2-7) | <0.001 | 4 (2-6) | <0.001 | 3 (1-5) | <0.001 |
| Mean score (SD) | 9.0 (3.5) | 6.2 (4.3) |  | 5.0 (3.4) |  | 4.3 (3.4) |  | 4.0 (3.8) |  |
| Pain on walking item |  |  |  |  |  |  |  |  |  |
| Median score (IQR) | 2 (1-2) | 1 (1-2) | <0.001 | 1 (0-1) | <0.001 | 1 (0-1) | <0.001 | 1 (0-1) | <0.001 |
| Mean score (SD) | 1.7 (1.0) | 1.3 (1.0) |  | 1.0 (0.8) |  | 0.8 (0.8) |  | 0.8 (0.9) |  |
| Stiffness subscale |  |  |  |  |  |  |  |  |  |
| Median score (IQR) | 4 (3-5) | NA | NA | 2 (1-3) | <0.001 | 2 (1-3) | <0.001 | 1 (0-2) | <0.001 |
| Mean score (SD) | 3.7 (1.7) | NA |  | 2.1 (1.5) |  | 1.9 (1.4) |  | 1.7 (1.7) |  |
| Functional capacity subscale |  |  |  |  |  |  |  |  |  |
| Median score (IQR) | 31 (24-37) | NA | NA | 17 (10-26) | <0.001 | 15 (7-22) | <0.001 | 13 (5-23) | <0.001 |
| Mean score (SD) | 31.0 (12.7) | NA |  | 19.3 (12.3) |  | 16.3 (11.8) |  | 15.1 (12.5) |  |
| **EQ-5D-5L** |  |  |  |  |  |  |  |  |  |
| Mobility |  |  |  |  |  |  |  |  |  |
| Median score (IQR) | 3 (2-3) | NA | NA | NA | NA | 2 (1-3) | <0.001 | NA | NA |
| Mean score (SD) | 2.8 (0.9) | NA |  | NA |  | 2.1 (0.8) |  | NA |  |
| Self-care |  |  |  |  |  |  |  |  |  |
| Median score (IQR) | 2 (1-3) | NA | NA | NA | NA | 1 (1-2) | <0.001 | NA | NA |
| Mean score (SD) | 2.2 (1.1) | NA |  | NA |  | 1.6 (0.8) |  | NA |  |
| Usual activities |  |  |  |  |  |  |  |  |  |
| Median score (IQR) | 3 (2-3) | NA | NA | NA | NA | 2 (1-2) | <0.001 | NA | NA |
| Mean score (SD) | 2.7 (0.9) | NA |  | NA |  | 2.0 (0.9) |  | NA |  |
| Pain/discomfort |  |  |  |  |  |  |  |  |  |
| Median score (IQR) | 3 (3-4) | NA | NA | NA | NA | 2 (2-3) | <0.001 | NA | NA |
| Mean score (SD) | 3.0 (0.8) | NA |  | NA |  | 2.2 (0.8) |  | NA |  |
| Anxiety/depression |  |  |  |  |  |  |  |  |  |
| Median score (IQR) | 2 (1-3) | NA | NA | NA | NA | 1 (1-2) | <0.001 | NA | NA |
| Mean score (SD) | 2.1 (1.1) | NA |  | NA |  | 1.5 (0.8) |  | NA |  |
| EQ VAS |  |  |  |  |  |  |  |  |  |
| Median score (IQR) | 60 (40-75) | NA | NA | NA | NA | 70 (60-85) | <0.001 | NA | NA |
| Mean score (SD) | 55.4 (22.4) | NA |  | NA |  | 69.2 (19.7) |  | NA |  |
| **Consumption of analgesics/NSAIDs as recue medication**, n (%) patients | 120 (72.3) | 90 (58.8) | <0.001^**^ | 83 (50.0) | <0.001^**^ | 63 (38.0) | <0.001^**^ | 58 (38.7) | <0.001^**^ |

WOMAC, Western Ontario and McMaster Universities Osteoarthritis Index; IQR, interquartile range (percentile 25-75); SD, standard deviation; NA, not available; VAS, visual analogue scale; NSAIDs, nonsteroidal anti-inflammatory drugs

* If not indicated otherwise, the statistical analysis was Wilcoxon test, ** McNemar test

**Supplementary Table 4**. Relative reductions of WOMAC results at all post-injection visits compared with baseline

|  | **2-week visit** | **4-week visit** | **12-week visit** | **24-week visit** |
| --- | --- | --- | --- | --- |
| N | 153 | 166 | 166 | 150 |
| **WOMAC questionnaire**, mean relative reduction (95% confidence interval) |  |  |  |  |
| Pain subscale | 33.4  (27.6-39.2) | 41.5  (35.2-47.8) | 48.2  (41.4-55.0) | 52.1  (44.3-59.9) |
| Pain on walking item | 24.7  (16.4-33.0) | 36.5  (28.0-45.0) | 47.1  (38.9-55.4) | 50.5  (41.3-59.7) |
| Stiffness subscale | NA | 40.1  (32.3-47.9) | 45.9  (39.0-52.9) | 49.1  (40.0-58.2) |
| Functional capacity subscale | NA | 33.4  (26.3-40.5) | 42.4  (36.0-48.9) | 47.4  (40.4-54.4) |

NA, not available

**Supplementary Table 5**. Change in WOMAC results at all post-injection visits compared with baseline

|  | **2-week visit** | **4-week visit** | **12-week visit** | **24-week visit** |
| --- | --- | --- | --- | --- |
| **PAIN SUBSCALE** |  |  |  |  |
| N | 153 | 166 | 166 | 150 |
| Patients who achieved an improvement, n (%) | 116 (75.8) | 140 (84.3) | 144 (86.7) | 131 (87.3) |
| Patients with no score change, n (%) | 23 (15.0) | 13 (7.8) | 6 (3.6) | 5 (3.3) |
| Patients whose disease worsened, n (%) | 14 (9.2) | 13 (7.8) | 16 (9.6) | 14 (9.3) |
| **PAIN ON WALKING** **ITEM** |  |  |  |  |
| N | 153 | 166 | 166 | 150 |
| Patients who achieved an improvement, n (%) | 69 (45.1) | 101 (60.8) | 102 (61.5) | 96 (64.0) |
| Patients with no score change, n (%) | 71 (46.4) | 49 (29.5) | 52 (31.3) | 43 (28.7) |
| Patients whose disease worsened, n (%) | 13 (8.5) | 16 (9.6) | 12 (7.2) | 11 (7.3) |
| **STIFFNESS** **SUBSCALE** |  |  |  |  |
| N | NA | 166 | 166 | 150 |
| Patients who achieved an improvement, n (%) | NA | 120 (72.3) | 124 (74.7) | 115 (76.7) |
| Patients with no score change, n (%) | NA | 31 (18.7) | 27 (16.3) | 19 (12.7) |
| Patients whose disease worsened, n (%) | NA | 15 (9.0) | 15 (9.0) | 16 (10.7) |
| **FUNCTIONAL CAPACITY SUBSCALE** |  |  |  |  |
| N | NA | 164 | 165 | 149 |
| Patients who achieved an improvement, n (%) | NA | 134 (81.7) | 140 (84.8) | 125 (83.9) |
| Patients with no score change, n (%) | NA | 9 (5.5) | 4 (2.4) | 5 (3.4) |
| Patients whose disease worsened, n (%) | NA | 21 (12.8) | 21 (12.7) | 19 (12.8) |

NA, not available

**Supplementary Table 6**. Calculations to analyze the minimal perceptible clinical improvement** with the treatment at the 12-week visit

|  | **Baseline** | **12-week visit** | **Absolute change** | **p *** |
| --- | --- | --- | --- | --- |
| **PAIN SUBSCALE**: 20.6 > 9.7** |  |  |  |  |
| Mean (SD) | 45.1 (17.5) | 21.2 (16.8) | 23.9 (21.6) | <0.001 |
| 95%CI | 42.5-47.8 | 18.7-23.8 | 20.6-27.2 |  |
| **PAIN ON WALKING ITEM**: 17.3 > 11.1** |  |  |  |  |
| Mean (SD) | 42.3 (24.4) | 20.9 (21.0) | 21.4 (26.7) | <0.001 |
| 95%CI | 38.6-46.1 | 17.7-24.2 | 17.3-25.5 |  |
| **STIFFNESS SUBSCALE**: 18.3 > 10.0** |  |  |  |  |
| Mean (SD) | 46.1 (21.8) | 23.9 (18.0) | 22.2 (25.3) | <0.001 |
| 95%CI | 42.8-49.4 | 21.1-26.6 | 18.3-26.1 |  |
| **FUNCTIONAL CAPACITY SUBSCALE**: 18.3 > 9.3** |  |  |  |  |
| Mean (SD) | 45.6 (18.6) | 24.0 (17.3) | 21.6 (21.4) | <0.001^***^ |
| 95%CI | 42.7-48.5 | 21.4-26.7 | 18.3-24.9 |  |

Values of WOMAC are transformed from Likert 0-20 to visual analogue scale 0-100

SD, standard deviation; 95%CI, 95% confidence interval

* If not indicated otherwise, the statistical analysis was Wilcoxon test

**Minimal perceptible clinical improvement was analyzed by following the Ehrich et al. methodology [19]

*** Paired samples t-test
